# Metabolomic analysis of children with congenital heart disease complicated by neurological developmental abnormalities and CHD7 mutations

**DOI:** 10.3389/fgene.2026.1885466

**Published:** 2026-08-28

**Authors:** Xianghui Huang, Wanting Li, Ying Lin, Dan Li, Huiyue Zhang, Yuandan Chen, Wei Sheng, Deyi Zhuang

**Affiliations:** 1 Children’s Hospital of Fudan University (Xiamen Branch), Xiamen Children’s Hospital, Fujian Key Laboratory of Neonatal Diseases, Xiamen Key Laboratory of Neonatal Diseases, Xiamen, China; 2 Jinjiang Municipal Hospital (Shanghai Sixth People’s Hospital Fujian), Jinjiang, China; 3 Children’s Hospital of Fudan University, Shanghai Key Laboratory of Birth Defects, Shanghai, China; 4 Xiamen Neonatal Quality Control Center, Xiamen, China

**Keywords:** CHD7 mutation, differential metabolites, left-to-right shunt congenital heart disease, metabolomics, neurodevelopmental abnormalities

## Abstract

**Objective:**

This study aimed to characterize the clinical features and identify serum differential metabolites in children with left-to-right shunt congenital heart disease (CHD) complicated by neurodevelopmental abnormalities (NDA) and harboring CHD7 mutations, to elucidate potential pathogenic mechanisms.

**Methods:**

A case-control study was conducted with three groups: seven children with CHD7-mutant CHD-NDA, 24 children with isolated CHD, and nine healthy controls. Serum metabolomic profiling was performed using untargeted liquid chromatography-tandem mass spectrometry (LC-MS/MS) in both positive and negative ion modes.

**Results:**

The metabolomic profiles showed a tendency toward separation among the CHD7-mutant CHD-NDA, isolated CHD, and healthy control groups. Candidate differential metabolites were mainly enriched in steroid hormone biosynthesis, glyoxylate and dicarboxylate metabolism, ascorbate and aldarate metabolism, and glutathione metabolism in the CHD7-mutant group compared with the isolated CHD group. Compared with healthy controls, the CHD7-mutant group also showed candidate alterations related to steroid hormone biosynthesis, riboflavin metabolism, and folate biosynthesis. Two overlapping candidate metabolites, 11-deoxycortisol and 2-hydroxyestrone, were identified across pairwise comparisons and may represent potential metabolic markers related to steroid metabolism.

**Conclusion:**

The metabolic disturbances observed in children with CHD7-associated CHD-NDA may be related to steroid metabolism and hypothalamic-pituitary axis regulation. These preliminary findings suggest a potential metabolic link between CHD7 mutations, cardiac phenotypes, and neurodevelopmental abnormalities, warranting further validation in larger, sex-matched cohorts.

## 1 Introduction

Congenital heart disease (CHD) is one of the most common congenital malformations, with a global incidence of approximately 8–10 per 1,000 live births, and remains a major contributor to congenital anomaly-related morbidity and mortality worldwide (Mires et al., 2024; Zhao, 2020). Although advances in diagnosis, perioperative care, and surgical treatment have substantially improved survival, neurodevelopmental disorders (NDDs) remain frequent long-term complications in children with CHD, affecting up to 50% of survivors (Nattel et al., 2017; Patt et al., 2023; Provost et al., 2024). These impairments may involve cognition, adaptive function, motor skills, language, attention, behavior, executive function, and autism spectrum-related manifestations (Nattel et al., 2017; Patt et al., 2023; Provost et al., 2024). Recent evidence indicates that neurodevelopmental outcomes in CHD are influenced by multiple interacting factors, including genetic susceptibility, fetal and perinatal conditions, altered cerebral oxygen delivery, perioperative factors, socioeconomic disadvantage, and parental psychological distress (Sood et al., 2024). Elucidating the pathogenesis of NDDs in this population is therefore critical for improving long-term outcomes.

Recent genetic studies have highlighted a close link between CHD and neurodevelopmental abnormalities. The chromodomain helicase DNA-binding protein 7 (CHD7) gene encodes a chromatin-remodeling protein involved in transcriptional regulation. Loss-of-function mutations in CHD7 are associated with several developmental disorders, including cardiac malformations, abnormal neuronal differentiation, and impaired forebrain development (Huang, 2023; Sta et al., 2023). Approximately 80% of individuals with CHD7 mutations present with congenital heart defects, often accompanied by craniofacial anomalies (Sta et al., 2023; Sun et al., 2022).

Metabolomics, as a high-throughput analytical approach, offers a powerful strategy to uncover biochemical alterations underlying CHD and associated neurodevelopmental impairments by profiling small-molecule metabolites (Mires et al., 2024; Bassareo and McMahon, 2022). This technique can capture complex gene-environment interactions, enabling more holistic prediction of phenotypic outcomes (Bassareo and McMahon, 2022). Several studies support the utility of metabolomics in diagnosing CHD and assessing prognosis (Vedovelli et al., 2019; Li et al., 2025; Cedars et al., 2021; Bahado-Singh et al., 2014; Yuan et al., 2022; Dong et al., 2025). For instance, liquid chromatography-mass spectrometry (LC-MS) analyses have revealed significant metabolic disturbances in CHD, identifying compounds such as N-acetylglucosamine-6-phosphate (GlcNAc-6-P), mannitol, and specific plasma cholines as potential biomarkers (Zhang, 2018). Nevertheless, few investigations have compared metabolic pathways or biomarkers between CHD patients with and without comorbid neurodevelopmental abnormalities.

Altered fetal hemodynamics and oxygenation patterns may contribute to abnormal metabolic profiles, thereby influencing neurodevelopment (Vedovelli et al., 2019; Andescavage et al., 2023). While right-to-left shunt lesions are linked to impaired cerebral oxygen delivery and cortical disruption, left-to-right shunt CHD—the most common subtype—typically lacks early hemodynamic or hypoxic challenges yet still demonstrates a considerable incidence of NDDs. Thus, understanding the mechanisms of neurodevelopmental impairment in left-to-right shunt CHD is of particular importance.

Neurodevelopment begins during the embryonic period, when forebrain structures crucial for energy metabolism, gene expression, and cellular growth are established (Liu et al., 2017). Cardiac development, which arises from neural crest cells and involves intricate signaling and metabolic pathways, is highly sensitive to disruption at any stage (Huang, 2009). It remains an important research question whether disturbances in early neural development through altered metabolism and signaling may subsequently affect cardiac morphogenesis.

A current frontier in this field involves integrating metabolomic data with genomic, transcriptomic, and epigenomic datasets to systematically decipher the molecular mechanisms by which CHD-associated genetic variants contribute to concurrent cardiac and neurodevelopmental abnormalities. Multi-omics approaches may reveal novel biomarkers, identify potential therapeutic targets, and improve risk stratification for children at high neurodevelopmental risk (Gao et al., 2024). Recent studies based on the developmental origins of health and disease hypothesis further suggest that placental dysfunction, fetal cardiac abnormalities, and early brain developmental disturbances may interact during embryonic and fetal life, thereby contributing to later neurodevelopmental disorders in children with CHD (22). Notably, Loblein et al. reported a higher prevalence of neurodevelopmental disorders, including attention-deficit/hyperactivity disorder (ADHD), autism spectrum disorder, and specific learning disorders, in a clinically referred cohort of children with CHD compared with population norms (Loblein et al., 2023). However, most previous metabolomic studies have focused on isolated cardiac phenotypes, fetal CHD prediction, or postoperative prognosis (Vedovelli et al., 2019; Li et al., 2025; Cedars et al., 2021; Bahado-Singh et al., 2014; Yuan et al., 2022; Dong et al., 2025). Whether CHD7 mutations contribute to concurrent cardiac and neurodevelopmental abnormalities through specific metabolic disturbances remains unclear. To our knowledge, no previous untargeted metabolomic study has systematically compared children with CHD7-mutant CHD complicated by neurodevelopmental abnormalities, children with isolated CHD, and healthy controls.

The present study aimed to use untargeted LC-MS/MS-based metabolomics to explore serum candidate differential metabolites and perturbed metabolic pathways in children with left-to-right shunt CHD, neurodevelopmental abnormalities, and CHD7 mutations. We sought to identify preliminary metabolic clues that may link cardiac phenotypes and neurodevelopmental impairment in this rare clinical subgroup, thereby providing a basis for future validation studies and mechanistic investigations.

## 2 Methods

### 2.1 Study population

Thirty-one children with left-to-right shunt CHD (7 with CHD7 mutations and neurodevelopmental abnormalities, 24 with isolated CHD) and nine healthy controls were enrolled. Cardiac phenotypes included ventricular septal defect (VSD), atrial septal defect (ASD), atrioventricular septal defect (AVSD), and patent ductus arteriosus (PDA) (Table 1). In the CHD7 mutation group, cardiac phenotypes included 3 cases of VSD, 2 cases of ASD, and 3 cases of AVSD. In the isolated CHD group, there were 10 cases of VSD, 6 cases of ASD, 2 cases of AVSD, and 6 cases of PDA.

**TABLE 1:** Clinical characteristics and systemic manifestations of the CHD7 group.

*CHD7* Case	Results of sequencing		Clinical phenotype										
	Variation	Clinical significance	Malform-ation of heart (81.3%)^*^	Hypothalam-ic pituitary dysfunction and genital abnormaliti-es (43.8%)^*^	Kidney/bo-ne/limb abnormalit-ies (46%–80%)^*^	Cranial nerve palsy or brain stem dysfuncti-on (87.5%)^*^	Abnormal brain structure (6.25%)^*^	Developm-ental delay/inte-llectual disability/autism (18.8%)^*^	Abnormal outer/mid-dle/inner -ear (93.8%)^*^	Malformat-ion of esophagus (6.25%)^*^	Posterior -nostril atresia (37.5%)^*^	Cleft palate (12.5%)^*^	Difficulty in swallowing/feeding (6.25%)^*^
Case 1	c.1951_1952 delAAinsT p.L651X	pathogenic	+	​	​	​	+	+	​	​	​	​	​
Case 2	c.4353 + 4_4353 + 12 delinsGCCCA	pathogenic	+	​	+	+	+	​	​	​	+	​	+
Case 3	c.3106C>T; p.A1036X	pathogenic	+	+	​	​	​	+	+	​	​	​	​
Case 4	c.2913C>G; p.Y971X	pathogenic	+	+	+	​	+	​	​	+	​	+	​
Case 5	c.8062dupA; p.I2688Nfs*3	pathogenic	+	​	​	+	​	​	+	​	​	​	​
Case 6	c.4386T>G; p.P1462E	Mutation:D SIFT:D/0.03 PolyPhen2:D/0.996	+	+	​	+	​	​	​	+	​	​	​
Case 7	c.2021C>G; p.P674R	Mutation:D SIFT:T/0.06 PolyPhen2:D/0.963	+	+	+	​	+	​	​	​	​	​	​

* indicates the reported prevalence of each anomaly in the literature (Cedars et al., 2021; Bahado-Singh et al., 2014). Pathogenic classifications follow ACMG, guidelines; SIFT, and PolyPhen2 scores indicate deleterious (D) or tolerated (T) effects.

Written informed consent was obtained from the guardians of all participants. The study was conducted in accordance with the Declaration of Helsinki, and ethical approval was obtained from the Medical Ethics Committee of Xiamen Children’s Hospital (approval No. [2024]29). Healthy controls were recruited from children who underwent routine health evaluation or preoperative examination for elective surgery during the same period and had no known congenital heart disease, neurodevelopmental disorder, acute infection, chronic systemic disease, or long-term medication exposure according to available clinical records. Fasting venous blood was obtained during routine clinical blood sampling after guardian consent.

### 2.2 Sample preparation

Sample preparation followed a standardized untargeted Liquid chromatography-tandem mass spectrometry (LC-MS/MS) metabolomics workflow (Broeckling et al., 2023). Fasting venous blood was collected and centrifuged at 3000 rpm for 10 min at 4 °C to obtain serum. Serum samples were stored at −80 °C until analysis.Metabolites were extracted using 80% methanol containing 0.1% formic acid. After vortexing and centrifugation at 15,000 rpm for 15 min at 4 °C, the supernatant was filtered through a 0.22 μm membrane filter. Quality control (QC) samples were prepared by pooling equal aliquots of all serum samples and were injected intermittently throughout the analytical sequence to monitor system stability and reproducibility.A detailed workflow of sample preparation is provided in Supplementary Table S1 to ensure methodological transparency and reproducibility.

### 2.3 LC-MS/MS conditions

Serum metabolomic analysis was performed by a commercial metabolomics service using an untargeted LC-MS/MS platform. Serum metabolites were extracted using organic solvent precipitation and analyzed by high-resolution LC-MS/MS. Data were acquired separately in electrospray ionization positive and negative ion modes. The analytical workflow included chromatographic separation, high-resolution MS/MS detection, QC monitoring, and database-assisted metabolite annotation. Raw data were processed using Compound Discoverer 3.1, and metabolite annotation was performed by matching accurate mass and MS/MS fragmentation patterns against public databases.

### 2.4 Data analysis

Raw LC-MS/MS data files (.raw) were imported into Compound Discoverer 3.1 for peak detection, peak alignment, retention-time correction, normalization, and compound annotation. Metabolite annotation was performed by matching accurate mass, isotope pattern, adduct information, retention characteristics, and MS/MS fragment information against mzCloud, ChemSpider, and mzVault databases. Features showing acceptable reproducibility in quality control (QC) samples were retained for subsequent statistical analysis.

Multivariate statistical analyses, including principal component analysis (PCA) and partial least squares-discriminant analysis (PLS-DA), were performed to explore overall metabolic differences among groups. Model interpretation was performed cautiously considering the small sample size of the CHD7-mutant cohort. Candidate differential metabolites were screened according to variable importance in projection (VIP) values from PLS-DA, fold-change criteria (FC > 1.2 or FC < 0.833), and nominal P values < 0.05. Given the exploratory nature of this rare disease cohort, these metabolites were considered candidate differential metabolites rather than validated biomarkers.

Univariate analysis was performed using SPSS 22.0 (IBM, Armonk, NY, USA). The normality of data distribution was assessed using the Shapiro–Wilk test. Because several variables were not normally distributed, the Mann–Whitney U test was applied for pairwise comparisons. Metabolites satisfying both multivariate and univariate criteria (VIP >1.0, FC > 1.2 or FC < 0.833, and nominal P < 0.05) were defined as candidate differential metabolites.

Pathway enrichment analysis was performed based on Kyoto Encyclopedia of Genes and Genomes (KEGG) annotation using candidate differential metabolites. Pathways with nominal P < 0.05 were considered potentially enriched. Given the small cohort size and the limited number of annotated metabolites in some pathways, pathway enrichment results were interpreted as exploratory and require further validation.

## 3 Results

### 3.1 General clinical data

There were no significant differences in gestational age, total protein, albumin, globulin, liver function, renal function, or other routine biochemical indices among the three groups (Table 2). The CHD7-mutant group consisted entirely of male patients, whereas the comparison groups included both males and females. This difference in sex distribution was noted as an important consideration for subsequent interpretation of steroid-related metabolic alterations.

**TABLE 2:** Clinical and biochemical characteristics of the three groups.

Clinical indicators	Control (n = 9)	CHD (n = 24)	*CHD7* (n = 7)
Sex (male)	n = 7	n = 16	n = 7
Gestational age	37.62 (5.45)	36.81 ± 6.45	37.25 ± 4.86
Total protein (g/L)	44.89 ± 13.43	48.16 ± 7.77	46.91 ± 11.10
Albumin (g/L)	29.53 ± 8.53	31.93 ± 4.81	31.29 ± 7.24
Globulin (g/L)	15.43 ± 5.13	16.23 ± 4.08	15.67 ± 4.10
White ball ratio	1.84 ± 0.59	2.06 ± 0.44	1.99 ± 0.48
Total bilirubin (μmol/L)	83.59 ± 50.45	86.86 ± 49.77	76.47 ± 47.06
Direct bilirubin (μmol/L)	17.77 ± 20.94	14.14 ± 17.02	13.69 ± 19.76
Indirect bilirubin (μmol/L)	67.53 ± 49.86	72.72 ± 48.22	63.52 ± 45.43
Direct bilirubin/Indirect bilirubin	0.26 ± 0.26	0.20 ± 0.17	0.22 ± 0.22
Glutamyltranspeptidase (U/L)	153.00 ± 88.71	180.39 ± 113.4	191.23 ± 96.98
Alkaline phosphatase (U/L)	193.59 ± 125.00	224.87 ± 109.04	202.42 ± 117.56
Glutamic-pyruvic transaminase (U/L)	15.50 ± 18.70	11.09 ± 14.00	12.29 ± 13.70
Glutamic-oxaloacetic transaminase (U/L)	47.26 ± 28.90	42.74 ± 26.01	52.61 ± 30.85
Glutamic-pyruvic transaminase/Glutamic-oxaloacetic transaminase	5.53 ± 3.99	6.50 ± 4.91	6.33 ± 3.34
Creatinine (mmol/L)	57.38 ± 22.48	55.84 ± 17.77	61.95 ± 21.04
Urea nitrogen (mmol/L)	5.07 ± 2.79	3.93 ± 2.28	4.62 ± 2.82
Total bile acid (umol/L)	15.37 ± 13.43	15.93 ± 19.63	13.16 ± 12.79
Cholinesterase (U/L)	4,688.52 ± 1401.43	5402.26 ± 1185.47	5119.37 ± 1298.26

Data are presented as mean ± standard deviation. All comparisons among groups were not statistically significant (P > 0.05).

### 3.2 Partial least squares-discriminant analysis (PLS-DA)

**FIGURE 1:**
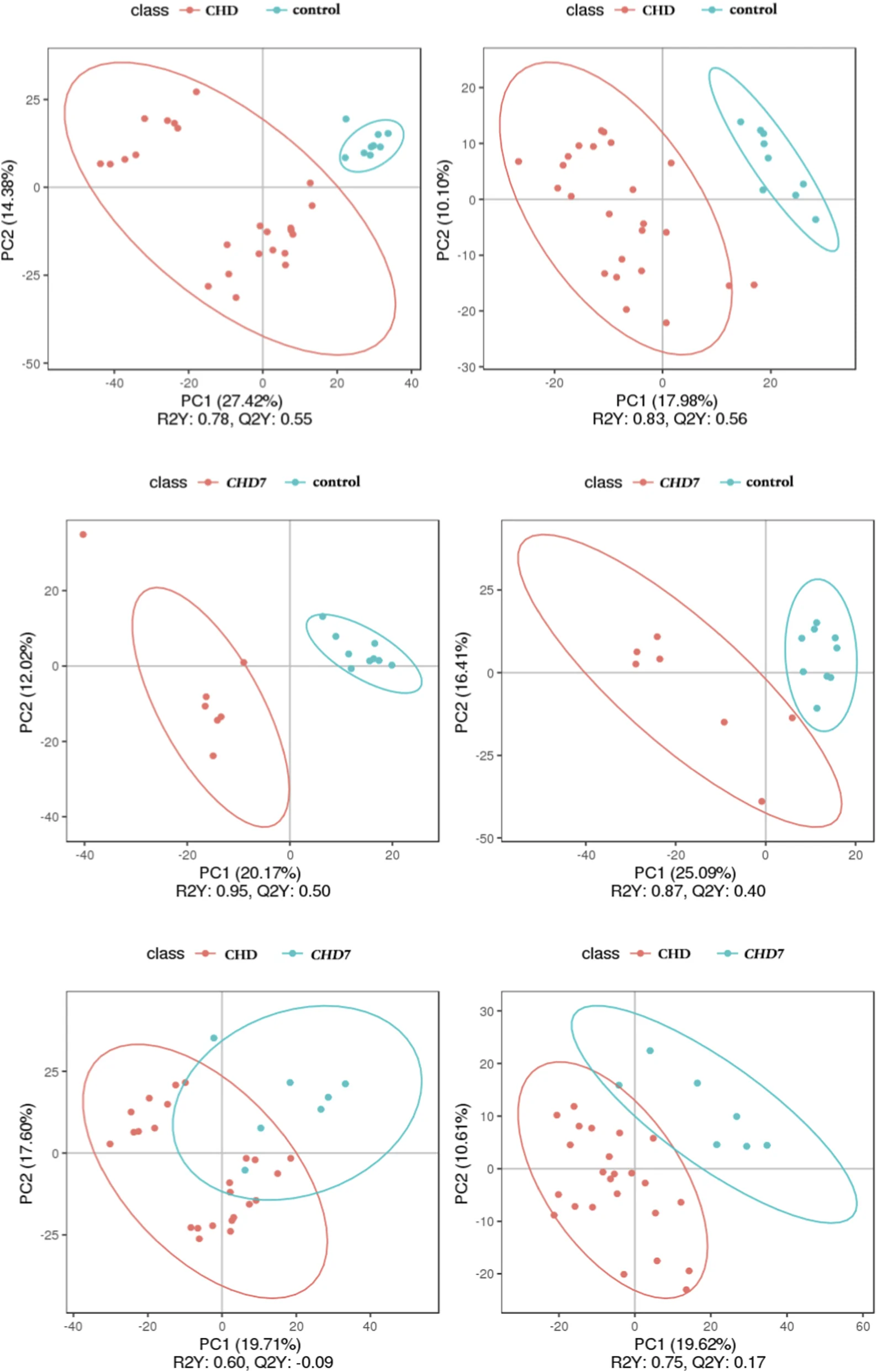
PLS-DA score plots of serum metabolic profiles among CHD7, CHD, and control groups. (Left: positive ion mode; Right: negative ion mode). Note: PLS-DA score plots showing serum metabolic profiles among CHD7, CHD, and control groups based on positive and negative ion modes. Left panels represent positive ion mode and right panels represent negative ion mode. R^2^Y indicates the explained variance of the model, and Q^2^Y indicates predictive ability. Given the limited sample size of the CHD7 group, these multivariate analyses were interpreted as exploratory.

PLS-DA score plots showed a tendency toward separation of metabolic profiles among the CHD7-mutant CHD-NDA, isolated CHD, and healthy control groups in both positive and negative ion modes (Figure 1). The separation between patient groups and healthy controls appeared more evident than that between the CHD7-mutant CHD-NDA and isolated CHD groups. Given the small sample size of the CHD7-mutant cohort, particularly for the CHD7-versus-CHD comparison, these multivariate findings should be interpreted cautiously and considered exploratory.

### 3.3 Volcano plot and cluster analysis

**FIGURE 2:**
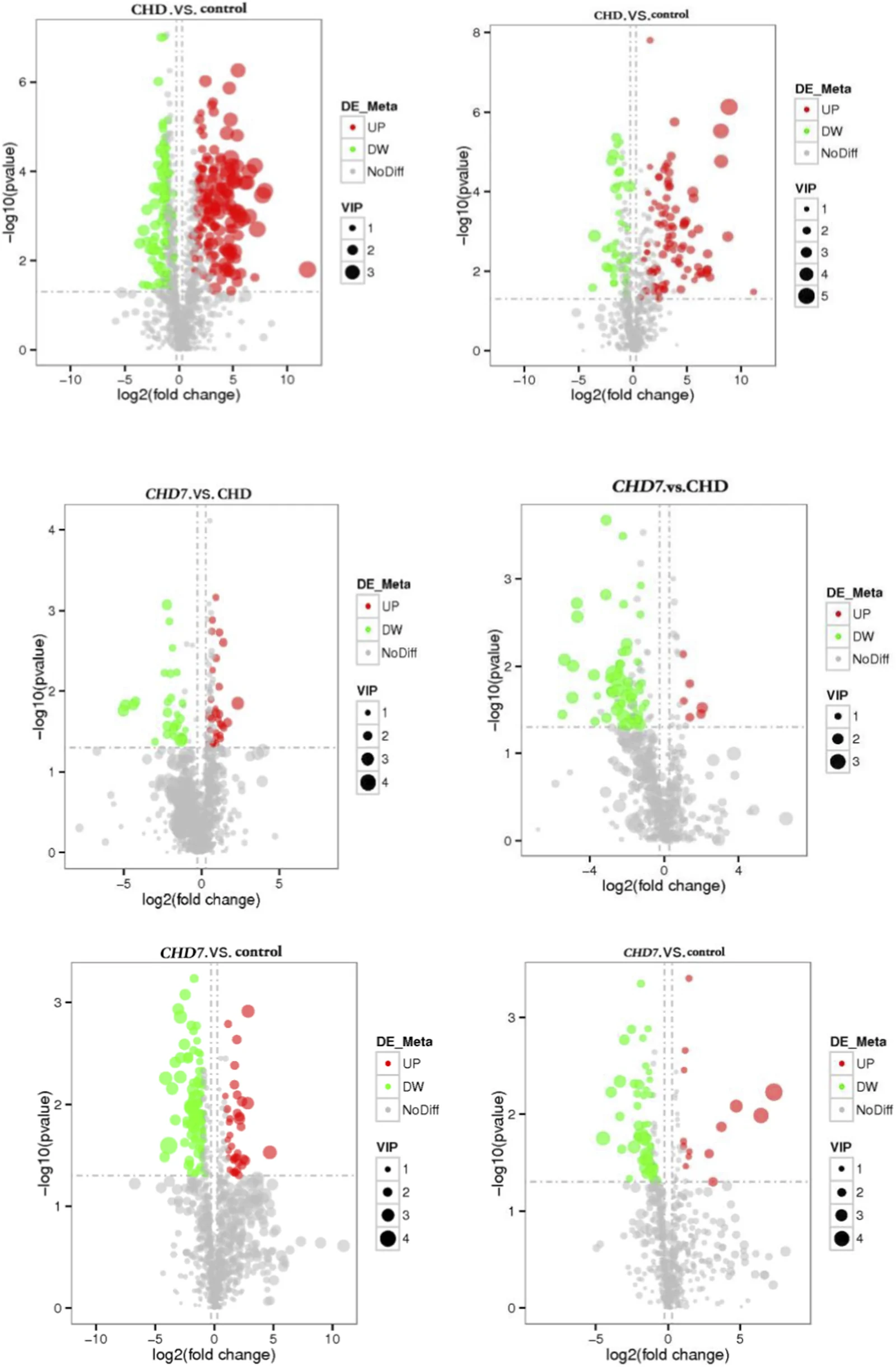
Volcano plots of candidate differential metabolites. (Left: positive ion mode; Right: negative ion mode). Note: Volcano plots illustrate candidate differential metabolites identified between groups based on VIP, fold change, and nominal P value criteria.

**FIGURE 3:**
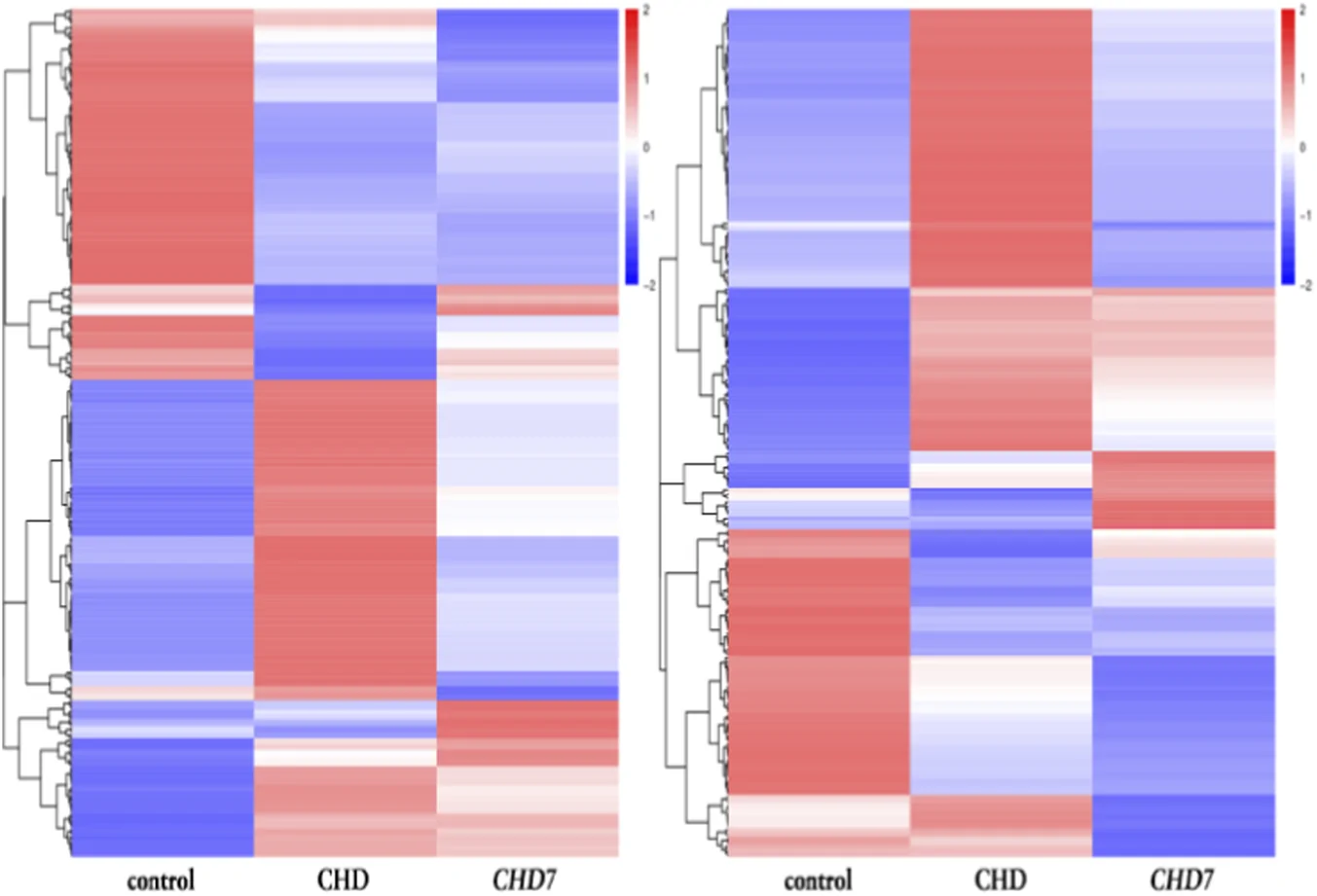
Hierarchical clustering analysis of candidate differential metabolites. (Left: positive ion mode; Right: negative ion mode). Note: Hierarchical clustering was performed using candidate differential metabolites identified from each comparison. The clustering patterns suggest differences in serum metabolic profiles among groups.

Volcano plots demonstrated multiple upregulated and downregulated candidate metabolites in both the CHD7-mutant CHD-NDA and isolated CHD groups compared with healthy controls (Figure 2). Hierarchical clustering analysis based on candidate differential metabolites showed distinct clustering patterns among groups, suggesting differences in serum metabolic profiles (Figure 3).

### 3.4 Identification of differential metabolites

**FIGURE 4:**
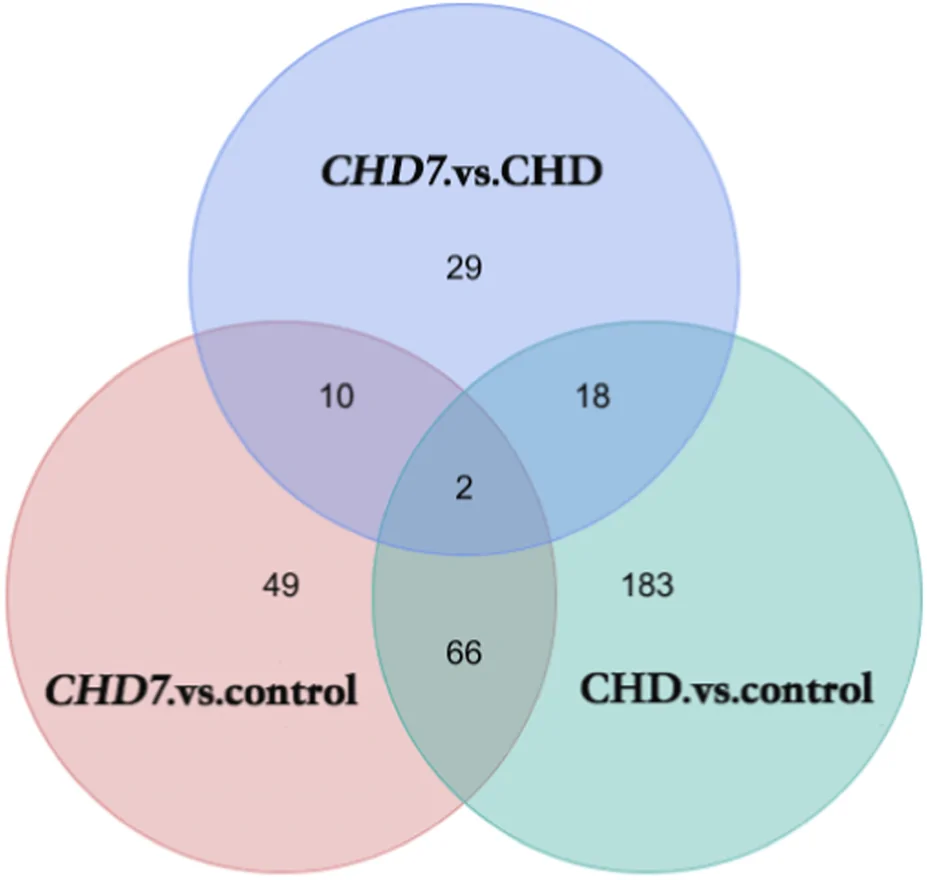
Venn diagram showing shared candidate metabolites across pairwise comparisons. Note: Venn diagram showing shared candidate metabolites identified among the three pairwise comparisons. Two overlapping candidate metabolites, 11-deoxycortisol and 2-hydroxyestrone, were observed across comparisons, suggesting shared steroid-related metabolic alterations among the studied groups.

Candidate differential metabolites were screened using the criteria VIP >1.0, FC > 1.2 or FC < 0.833, and nominal P < 0.05. The numbers of candidate differential metabolites identified in each comparison are summarized in Table 3. Venn diagram analysis identified two overlapping candidate metabolites, 11-deoxycortisol and 2-hydroxyestrone, among the three pairwise comparisons. These metabolites may represent candidate metabolic signals associated with steroid metabolism (Figure 4).

**TABLE 3:** Numbers of candidate differential metabolites identified in each comparison under positive and negative ion modes.

Comparison group	Total number of detected metabolites	Number of candidate differential metabolites	Number of upregulated candidate metabolites	Number of downregulated candidate
*CHD7*.vs.CHD (positive ion mode)	1219	59	30	29
*CHD7*.vs.CHD (negative ion mode)	696	60	6	54
*CHD*.vs.control (positive ion mode)	1219	269	163	106
*CHD*.vs.control (negative ion mode)	696	146	102	44
*CHD7*.vs.control (positive ion mode)	1219	127	37	90
*CHD7*.vs.control (negative ion mode)	696	65	14	51

Metabolomic features detected in positive and negative ion modes were analyzed separately. Candidate differential metabolites were screened based on VIP >1.0, fold change (FC) > 1.2 or FC < 0.833, and nominal P < 0.05. The numbers of upregulated and downregulated candidate differential metabolites are summarized for each pairwise comparison.

### 3.5 Metabolic pathway analysis

**FIGURE 5:**
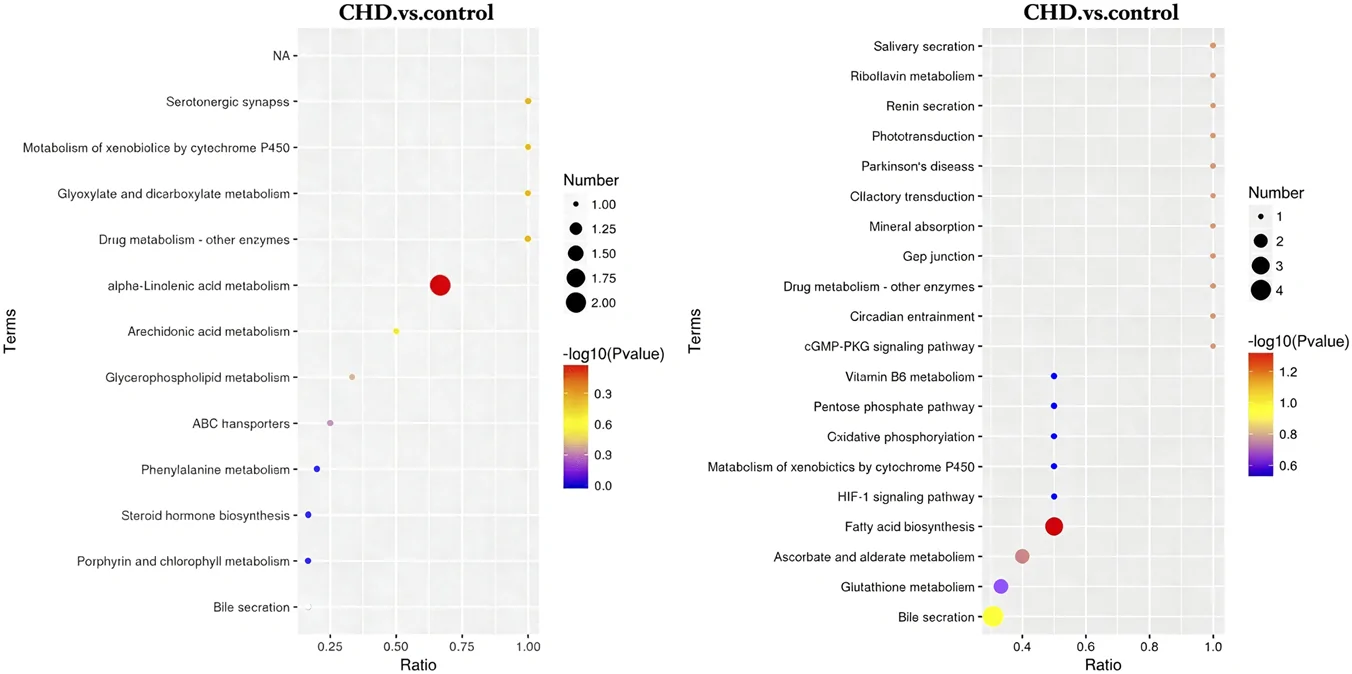
KEGG pathway enrichment analysis: CHD vs. Control. (Left: positive ion mode; Right: negative ion mode). Note: KEGG pathway enrichment analysis was performed based on candidate differential metabolites identified in each pairwise comparison. Pathways with nominal P < 0.05 were considered potentially enriched. The size of the dots represents the number of mapped metabolites.

**FIGURE 6:**
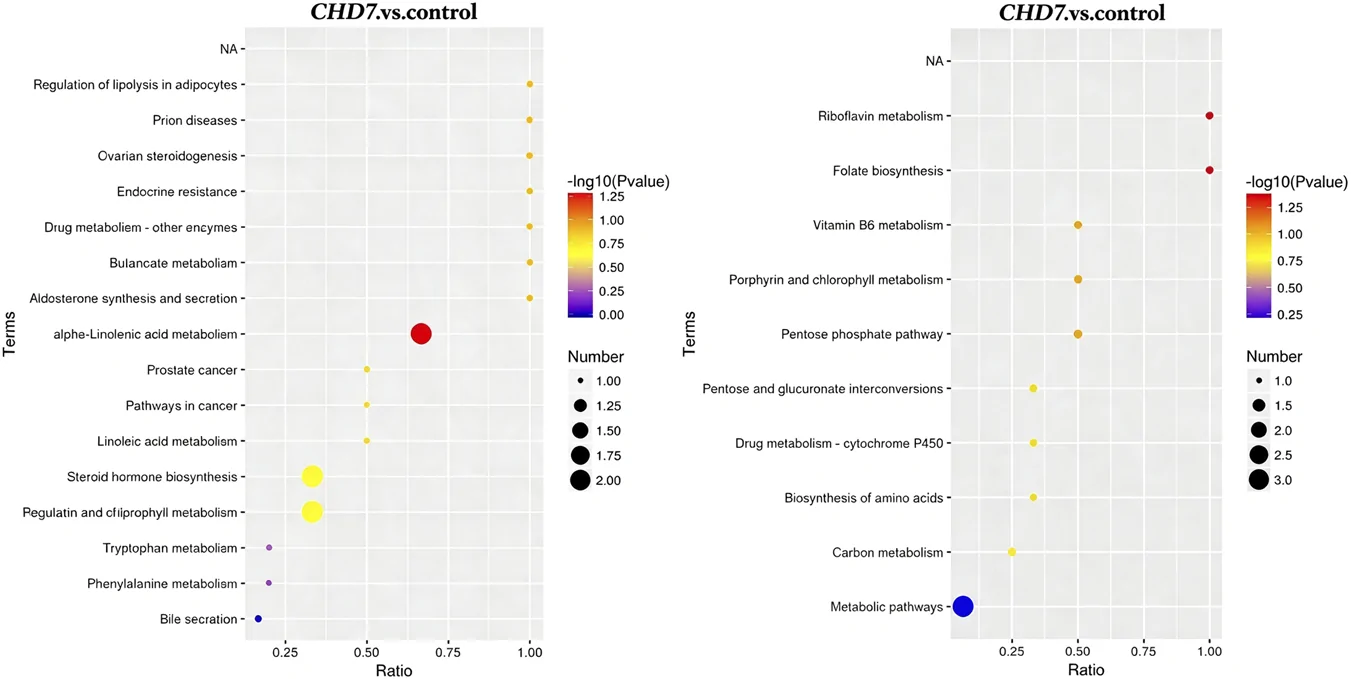
KEGG pathway enrichment analysis: CHD7 vs. Control. (Left: positive ion mode; Right: negative ion mode). Note: KEGG pathway enrichment analysis was performed based on candidate differential metabolites identified in each pairwise comparison. Pathways with nominal P < 0.05 were considered potentially enriched. The size of the dots represents the number of mapped metabolites.

**FIGURE 7:**
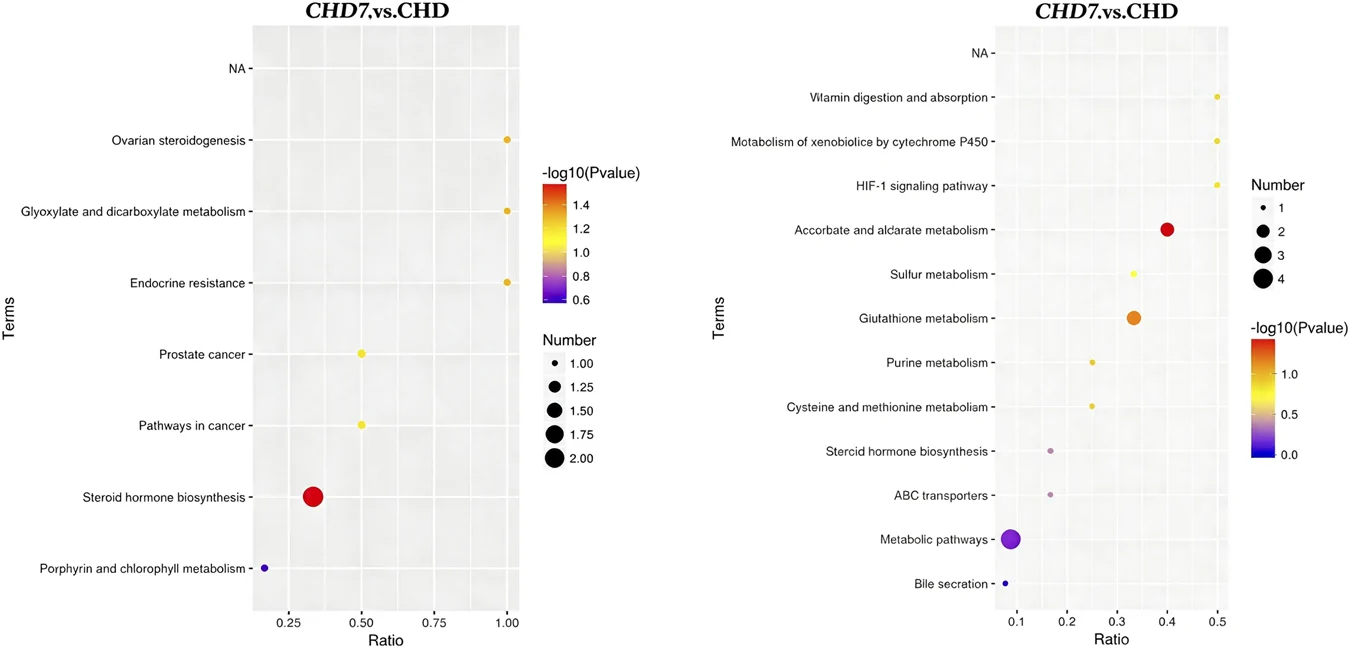
KEGG pathway enrichment analysis: CHD7 vs. CHD. (Left: positive ion mode; Right: negative ion mode). Note: KEGG pathway enrichment analysis was performed based on candidate differential metabolites identified in each pairwise comparison. Pathways with nominal P < 0.05 were considered potentially enriched. The size of the dots represents the number of mapped metabolites.

KEGG pathway enrichment analysis suggested several potentially enriched pathways among the candidate differential metabolites (Table 4; Figures 5–7). In the CHD7 vs. CHD comparison, potentially enriched pathways included steroid hormone biosynthesis, glyoxylate and dicarboxylate metabolism, ascorbate and aldarate metabolism, and glutathione metabolism (Table 4; Figure 5). In the CHD7 vs. control comparison, potentially enriched pathways included steroid hormone biosynthesis, riboflavin metabolism, and folate biosynthesis (Table 4; Figure 6). In the CHD vs. control comparison, potentially enriched pathways included alpha-linolenic acid metabolism and fatty acid biosynthesis (Table 4; Figure 7).

**TABLE 4:** KEGG pathway enrichment analysis of candidate differential metabolites.

Groups	Metabolic pathway	Candidate differential	All metabolites	P
*CHD7* vs. CHD	Steroid hormone biosynthesis	4	82	0.025
	Glyoxylate and dicarboxylate metabolism	4	82	0.049
	Ascorbate and aldarate metabolism	6	90	0.034
	Glutathione metabolism	6	90	0.049
*CHD7*vs control	Steroid hormone biosynthesis	12	82	0.045
	Riboflavin metabolism	4	90	0.044
	Folate biosynthesis	4	90	0.044
CHD vs. control	Alpha-linolenic acid metabolismα	13	82	0.041
	Fatty acid biosynthesis	14	90	0.046

Pathways with nominal P < 0.05 were considered potentially enriched.

## 4 Discussion

Untargeted metabolomics enables comprehensive profiling of small-molecule metabolites and provides a useful strategy for investigating disease-associated metabolic alterations (Broeckling et al., 2023). In this study, serum metabolic profiles were investigated in children with CHD7-mutant CHD-NDA, isolated CHD, and healthy controls. Candidate differential metabolites showed potential enrichment in steroid hormone biosynthesis-related pathways, including 11-deoxycortisol and 2-hydroxyestrone. These findings suggest that steroid-related metabolic alterations may be involved in CHD7-associated cardiac and neurodevelopmental phenotypes. However, considering the exploratory design, limited sample size, and lack of independent validation, these findings should be interpreted as preliminary metabolic signals rather than definitive biomarkers.

Steroid hormones participate in multiple biological processes, including energy metabolism, cellular proliferation, and tissue development (Matsuyama and DeFalc, 2024). CHD7 has been implicated in neural development and pituitary function through interactions with developmental regulatory pathways, including SRY-box transcription factor 2(SOX2) (Janssen et al., 2012; Gregory et al., 2013). The interpretation of estrogen-related metabolites requires caution. In the present study, all children in the CHD7 group were male, whereas the comparison groups included both males and females. Therefore, differences in sex distribution may have influenced steroid- and estrogen-related metabolic profiles, including 2-hydroxyestrone. Although this metabolite may provide preliminary clues regarding steroid metabolism, further validation in sex-matched cohorts is required before drawing conclusions regarding estrogen-mediated cardioprotective or developmental mechanisms.Therefore, the steroid-related metabolic signals observed in this study may reflect a potential association between CHD7-related developmental abnormalities and endocrine metabolic regulation.Although steroid hormone biosynthesis emerged as a potentially enriched pathway, these pathway enrichment findings should be interpreted cautiously because some pathways were represented by only a limited number of mapped metabolites. Therefore, these findings should be considered exploratory and require validation using targeted metabolomics and larger independent cohorts.However, because serum samples were collected after birth, these findings cannot establish whether steroid metabolic alterations directly contribute to embryonic cardiac development or neurodevelopmental abnormalities.

We found reduced 11-deoxycortisol in the CHD7 group, suggesting impaired glucocorticoid synthesis due to pituitary dysfunction. Glucocorticoids modulate cardiomyocyte survival, nitric oxide synthase activity, and inflammatory responses (Wang, 2020). Decreased 11-deoxycortisol may also disrupt cholesterol homeostasis, which is critical for neuronal dendrite formation, synaptic plasticity, and brain development (Yu et al., 2021). These changes may simultaneously impair cardiac development and neurodevelopment.

Downregulated 2-hydroxyestrone indicates reduced estrogen synthesis. Estrogen activates the Akt signaling pathway in cardiomyocytes to inhibit apoptosis and exerts cardioprotective effects (Patten and Karas, 2006). Estrogen also regulates Notch signaling in cardiac neural crest cells, which is essential for outflow tract and great vessel development (Barta et al., 2018; Serrano et al., 2019). Estrogen deficiency may therefore weaken cardioprotection and disrupt cardiac morphogenesis.Taken together, these findings suggest that steroid metabolic dysregulation may represent a shared biochemical pathway linking CHD7-associated developmental abnormalities with both cardiac and neurological phenotypes. Because CHD7 functions as an epigenetic regulator during embryogenesis, altered steroid metabolism may be one component of a broader molecular network involving endocrine regulation, oxidative stress, mitochondrial metabolism, and transcriptional remodeling (Sta et al., 2023; Gao et al., 2024; Demonceaux et al., 2025). Future studies integrating metabolomics with genomics, transcriptomics, and epigenomics are therefore warranted to further clarify how CHD7 mutations perturb heart–brain developmental programs.

From a clinical perspective, the identification of 11-deoxycortisol and 2-hydroxyestrone as shared differential metabolites suggests that steroid-related metabolites may serve as candidate biomarkers for early risk stratification. Combining genetic testing with metabolomic profiling may help identify children with CHD who are at increased risk for neurodevelopmental abnormalities and who may benefit from closer developmental surveillance and early intervention. The 2024 American Heart Association scientific statement emphasizes updated neurodevelopmental risk categories and recommends systematic referral, evaluation, and management for individuals with CHD at increased neurodevelopmental risk (Sood et al., 2024). Another consideration is that the CHD7 group represented a clinically heterogeneous multisystem cohort. Differences in nutritional status, feeding difficulty, growth status, medication exposure, and syndromic burden may also influence serum metabolic profiles. Therefore, some metabolic alterations observed in this study may reflect overall disease burden rather than CHD7-specific mechanisms.

This study has several limitations. First, the CHD7 group was relatively small because of the rarity of this condition, which may limit statistical power and generalizability. Second, this was a single-center cross-sectional study; therefore, causal relationships between CHD7 mutations, steroid-related metabolic alterations, and neurodevelopmental abnormalities cannot be established. Third, the metabolomic findings were exploratory, and candidate metabolites and enriched pathways require further validation using targeted metabolomics, authentic standards, improved statistical validation, and independent cohorts. Fourth, all children in the CHD7 group were male, and the clinical heterogeneity of CHD7-related disorders may have influenced steroid-related metabolic findings. Future multicenter studies incorporating sex-matched cohorts, endocrine assessment, longitudinal neurodevelopmental evaluation, and multi-omics approaches are warranted to validate and extend these findings.

## 5 Conclusion

In conclusion, this exploratory serum metabolomics study identified candidate metabolic alterations in children with CHD7-mutant CHD-NDA, with potential involvement of steroid hormone biosynthesis-related pathways. Candidate metabolites, including 11-deoxycortisol and 2-hydroxyestrone, may provide preliminary clues regarding metabolic characteristics associated with CHD7-related cardiac and neurodevelopmental phenotypes. However, given the small sample size, sex imbalance, exploratory statistical analysis, and database-based metabolite annotation, these findings require further validation in larger, sex-matched cohorts using targeted metabolomics, endocrine assessment, and longitudinal follow-up.

## Data Availability

The original contributions presented in the study are included in the article/Supplementary Material, further inquiries can be directed to the corresponding authors.
